# Value of quantitative analysis of left ventricular systolic function in patients on maintenance hemodialysis based on myocardial work technique

**DOI:** 10.1186/s12872-021-01899-6

**Published:** 2021-02-06

**Authors:** Chang Liu, Yi-Ping Feng, Zi-Ning Yan, Li Fan, Yi-Fei Rui, Ling Cui

**Affiliations:** Department of Echocardiography, The Affiliated Changzhou No. 2 People’s Hospital of Nanjing Medical University, Changzhou, 213003 China

**Keywords:** Echocardiography, Maintenance hemodialysis, Myocardial work, Left ventricular hypertrophy, Systolic function

## Abstract

**Background:**

This study aimed to determine the left ventricular (LV) systolic function in patients on maintenance hemodialysis (MHD) using the myocardial work (MW) technique and investigate the clinical value of the MW technique for the quantitative analysis of left ventricular (LV) systolic function in MHD patients with left ventricular hypertrophy (LVH).

**Methods:**

A total of 68 MHD patients and 35 controls were registered in this study. The MHD patients were divided into the non-left ventricular hypertrophy (NLVH) group (n = 35) and the LVH group (n = 33) according to the LV mass index (LVMI). MW was used to generate the LV global longitudinal strain (GLS), global work index (GWI), global constructive work (GCW), and global wasted work (GWW), global work efficiency (GWE). GLS and the MW parameters (GWI, GCW, GWW, GWE) were compared between groups and the correlations between these parameters and the LV ejection fraction (LVEF) in the LVH group were examined. The receiver operating characteristic (ROC) curve was used to evaluate the efficacy of MW parameters and GLS for the assessment of LV systolic dysfunction in MHD with LVH patients.

**Results:**

The LVH group had significantly lower GWE, GWI, GCW, and GLS but higher GWW than the control and NLVH groups. Compared with the control group, the NLVH group had significantly lower GWE and GLS and higher GWW, but no significant differences in GWI, GCW were observed between these two groups. The LVEF was negatively correlated with GWW in MHD patients, but positively correlated with GWI, GWE, and GCW in the LVH group. Receiver operating characteristic curve (ROC) analysis revealed that GWE, GWW, GWI, and GCW had appreciable area under the curve (AUC), sensitivity, and specificity for evaluating LV function in LVH patients on MHD.

**Conclusions:**

The MW parameters can quantitatively represent the LV myocardial work in MHD patients. Thus, the technique provides a new method for the quantitative evaluation of LV systolic function in MHD with LVH patients.

## Background

Left ventricular (LV) systolic and diastolic dysfunction, LV hypertrophy (LVH) and myocardial fibrosis are the main features of cardiomyopathy in patients with end-stage renal disease (ESRD) [1, 2]. Maintenance hemodialysis (MHD) is the mainstay of treatment for ESRD patients. However, Lagies et al. [3] suggested that the degree of LVH and LV systolic dysfunction continue to worsen despite the prolongation of hemodialysis time and that MHD is unable to completely improve LV myocardial damage. LVH is the main cardiovascular disease in MHD patients due to changes in the workload of the heart [4, 5] and is a strong predictor of cardiovascular morbidity and mortality in this population [6]. Thus, early detection and diagnosis of LV dysfunction as well as LVH in MHD patients are of great significance for the administration of proper treatments, thus reducing mortality.

Two-dimensional speckle tracking imaging (2D-STI) technology is an effective tool for evaluating LV systolic function [7]; it can semi-automatically analyze LV myocardial strain, which sensitively reflects changes in LV function and is an appreciable prognostic indicator of cardiac adverse events. However, 2D-STI is load-dependent, i.e. it cannot explain how load changes affect the LV myocardial strain [8]. For example, increased afterload may cause lower LV strain, leading to misjudgment of the real LV systolic function [9]. Myocardial work (MW), which is derived from 2D-STI, combines the LV pressure (derived from the systolic pressure in the brachial artery) measured using a noninvasive method and the longitudinal strain data for the LV myocardium to establish the LV pressure-strain loops (PSL) [10]. This approach can be used to examine the changes in LV MW when LV becomes deformed due to overcoming the afterload.

Currently, MW is used in the evaluation of the effects of cardiac resynchronization therapy on hypertension, primary cardiomyopathy, and coronary heart disease (CHD) [11, 12]. There are few reports on the use of MW in the evaluation of LV function in MHD patients. This study aimed to evaluate LV MW in patients with MHD. Our findings provide direct evidence of a new evaluation method for the early diagnosis of myocardial dysfunction in MHD patients.

## Methods

### Ethics statement

This study was approved by the Ethics Committee of Changzhou Second People’s Hospital affiliated with Nanjing Medical University(Changzhou, China). The informed consent form was signed by all participants.

### Participant selection

A total of 68 MHD patients with ESRD who were treated at the Second People’s Hospital of Changzhou between January 2019 and November 2020 were retrospectively enrolled in this study. The inclusion criteria were as follows: (1) the primary disease was kidney disease and was diagnosed as ESRD; (2) all patients had a forearm arteriovenous anastomosis fistula and had dialysis thrice a week for 4 hours each time, which lasted for 6–36 months; and (3) patients did not have the following diseases: heart valve disease, CHD, congenital heart disease, primary cardiomyopathy, arrhythmia, and pericardial effusion. According to the LV mass index (LVMI) [13], the 68 patients were divided into two groups: the non-left ventricular hypertrophy group (NLVH) (n = 35), and the LVH group (n = 33). Thirty-five healthy subjects with matching age and sex who had no history of heart disease, hypertension, abnormal liver and kidney function, and diabetes were enrolled in the control group. All controls had normal physical indexes, electrocardiograms, and echocardiograms.

### Data collection

The demographic and baseline clinical data for all participants were obtained from interviews and the hospital database.

### Conventional echocardiography

All participants underwent conventional two-dimensional Doppler echocardiography (Vivid E9, GE Healthcare, Horten, Norway). MHD patients were examined within 30 minutes after hemodialysis and brachial artery blood pressure was recorded. The following echocardiographic parameters were also recorded: LV internal diameter at end diastole (LVIDD), LV internal diameter at end systole (LVIDS), interventricular septal diameter (IVSD), LV posterior wall diameter (LVPWD), and left atrium diameter (LAD). Left ventricular mass (LVM) was calculated as follows: LVM = 0.8 × 1.04 × [(LVIDD + LVPWD + IVSD) ^3^ - LVIDD^3^] + 0.6, body surface area (BSA) = 0.0061 × height + 0.0128 × weight − 0.1529. LVM index (LVMI) = LVM / BSA. Blood flow velocity (E and A) in the early and late diastolic mitral orifice was determined using pulsed Doppler and the E/A ratio was calculated. The peak speed (e’) at the early mitral annulus motion was determined and the E/e’ ratio was calculated. Images were acquired for offline analysis: the subject was connected to the electrocardiogram with an image frame rate of 60–90 frames per second. The blood flow spectrum for the mitral valve and aortic valve ostium was collected, and the two-, three-, and four-chamber views during three consecutive cycles were stored on the hard disk.

### Automated function imaging (AFI)

The dynamic images of LV obtained from two-, three-, and four-chamber views as well as the images of blood stream spectrum from the mitral and aortic valves were imported into GE’s offline cardiac analysis software (EchoPac PC version 203, GE Healthcare, Horten, Norway). After determining the opening and closing times of the mitral and aortic valves on the spectrum images, AFI was used to sequentially delineate the LV endocardial system in the three-, four-, and two-chamber views, and automatically depicted the LV myocardium as a region of interest (ROI). We then manually adjusted the envelope curve to match the wall of the chamber. The system automatically provided the longitudinal strain curve for the entire LV including 17 LV segments, as well as the LV global longitudinal strain (GLS) and peak strain dispersion (PSD). The following two-plane LV volume parameters were obtained by pressing the ejection fraction (EF) button: LV end diastolic volume (LVEDV), LV end systolic volume (LVESV), and LVEF. The formula for LVEF was (LVEDV - LVESV)/ LVEDV × 100%.

GLS and several MW parameters, namely global work index (GWI), global constructive work (GCW), global wasted work (GWW), and global work efficiency (GWE), were obtained through the following sequential steps: (1) clicking the “myocardial work” button, (2) input of brachial artery blood pressure value, and (3) clicking the “Advanced” button. The system constructed an LV PSL based on the isovolumic contraction, ejection phase, and isovolumic relaxation phases, defined by the valve opening and closing times.

### Evaluation of intra- and inter‐observer variability

We randomly selected 30 subjects from the control group, the NLVH group, and the LVH group and compared the differences between two observations made by the same observer after one week. The differences between two independent observers who were unaware of the patient groupings were also evaluated. Interclass correlation coefficients (ICCs) were used to evaluate the intra- and inter-observer variability. The criteria for ICCs were: “excellent” if ≥ 0.80, “good” if 0.61–0.79, “moderate” if 0.41–0.60, and “poor” if ≤ 0.40.

### Statistical analysis

SPSS 22.0 (IBM SPSS, Statistics, Chicago, IL, USA) statistical software was used for all statistical analyses. The measurement data are expressed as mean ± standard deviation (SD), and the count data are expressed as n (%). Data normality was tested using Kolmogorov-Smirnov’s method. The single-factor analysis of variance was used for the comparison of normally distributed data among three groups, while the Student–Newman–Keuls (SNK) method was used to compare data between two groups. The Kruskal-Wallis method was used to compare non-normally distributed data. The rates in the general data were compared using the chi-square test or corrected chi-square test. *P* < 0.05 was considered statistically significant. For determination of the correlation between LVEF and the MW parameters including GWI, GWE, GCW, and GWW in MHD with LVH patients, Pearson correlation analysis was used for normally distributed variables, and Spearman correlation analysis was used for non-normally distributed variables. The receiver operating characteristic curve (ROC) was used to analyze the diagnostic efficacy of GWE, GWI, GCW, GWW, and GLS for LV systolic function in MHD with LVH patients. The Youden index was used to calculate the most appropriate cutoff for each value and to obtain the specificity and sensitivity. The criteria for the areas under the curves (AUCs) were: “good” if 0.90–1.00, “moderate” if 0.71–0.89, “poor” if 0.51–0.70, and “useless” if ≤ 0.50.

## Results

### Demographic and baseline clinical characteristics of participants

We first compared the demographic and baseline clinical characteristics of participants in the control group (n = 35; men: 19; women: 16), the NLVH group (n = 35; men: 18, LVMI ≤ 115 g/m^2^; women: 17, LVMI ≤ 95 g/m^2^), and the LVH group (n = 33; men: 19, LVMI > 115 g/m^2^; women: 14, LVMI > 95 g/m^2^). As shown in Table 1, no significant differences were observed with regard to age, sex, heart rate, and body mass index (BMI) between the groups (all *P* > 0.05). Further, there were no significant differences in remove volume, dry weight, cause of disease, and medication history between these three groups. As expected, the NLVH and LVH groups had significantly higher systolic blood pressure (SBP), diastolic blood pressure (DBP), and creatinine levels than the control group (*P* < 0.05), but no significant differences in these parameters were observed between the NLVH and LVH groups (*P* > 0.05). The N-terminal pro-brain natriuretic peptide (NT-proBNP) values ​​in the NLVH group and LVH group were significantly higher than those in the control group, and the NT-proBNP levels in the LVH group were significantly higher than those in the NLVH group. In addition, the LVH group had a significantly longer duration of dialysis than the NLVH group (*P* < 0.05).

**Table 1:** Demographic and baseline clinical characteristics of participants

	Controls (n = 35)	NLNH (n = 35)	LVH (n = 33)	*P* value
Age (years)	51.34 ± 8.10	52.11 ± 7.95	51.94 ± 7.50	0.913
Male sex (number)	19 (54.3 %)	18 (51.4 %)	18 (54.5 %)	0.959
Heart rate (beats/min)	71.77 ± 9.86	75.66 ± 7.92	75.94 ± 9.23	0.069
BMI (kg/m^2^)	22.51 ± 2.20	22.17 ± 1.95	22.09 ± 1.76	0.651
HD time (months)		12 ± 3	27 ± 5^#^	**< 0.001**
Remove volume (kg)		1.88 ± 1.00	52.55 ± 8.34	0.143
Dry weight (kg)		50.80 ± 7.93	2.21 ± 0.80	0.380
SBP (mmHg)	120.06 ± 5.72	146.06 ± 23.98*	148.18 ± 15.86*	**< 0.001**
DBP (mmHg)	69.43 ± 5.94	86.40 ± 10.63*	89.64 ± 9.16*	**< 0.001**
Creatinine (µmol/L)	63.86 ± 9.44	821.89 ± 182.04*	877.15 ± 177.15*	**< 0.001**
NT-proBNP (pg/ml)	66.74 ± 22.18	1212.06 ± 419.68*	14551.06 ± 10831.02^*#^	**< 0.001**
NYHA II-IV (cases)		8 (23 %)	32 (97 %)^#^	**< 0.001**
Cause of ESRD				
Glomerulonephritis (cases)		17 (48.6 %)	17 (51.5 %)	0.808
Diabetic Nephropathy (cases)		9 (25.7 %)	7 (21.2 %)	0.662
Hypertensive nephrosclerosis (cases)		4 (11.4 %)	4 (12.1 %)	0.929
Polycystic kidney (cases)		3 (8.6 %)	2 (6.1 %)	0.692
Unknown (case)		2 (5.7 %)	3 (9.1 %)	0.594
Medication				
ACEI/ARB (case)		18 (51 %)	21 (64 %)	0.309
Calcium channel blockers (case)		22 (63 %)	24 (73 %)	0.385
β-Receptor blockers (case)		14 (40 %)	16 (49 %)	0.481

Data are expressed as mean ± SD or number of subjects (%). Bold number: *P* < 0.05. *P < 0.05, vs. normal. ^#^*P* < 0.05, vs. NLVH. BMI, body mass index; HD, hemodialysis; SBP, systolic blood pressure; DBP, diastolic blood pressure; NT-proBNP, N-terminal pro-brain natriuretic peptide; ESRD: end-stage renal disease

### Conventional echocardiographic parameters for participants

We next compared the conventional echocardiographic parameters between these three groups. Compared with the control group, the NLVH and LVH groups had significantly higher LVIDD, LVIDS, LAD, LVEDV, and LVESV (*P* < 0.05), but there were no significant differences in these parameters between the NLVH and LVH groups (P > 0.05). In addition, the LVH group had a significantly lower LVEF than the control group (P < 0.05). The LVH group had significantly worse E/A and E/e’ than the control and NLVH groups, while the NLVH group had significantly worse E/A and E/e’ than the control group, indicating that MHD patients, especially those in the LVH group, had diastolic dysfunction (Table 2).

**Table 2:** Conventional echocardiographic parameters for participants

	Controls (n = 35)	NLVH (n = 35)	LVH (n = 33)	*P* value
LVIDD (mm)	46.17 ± 3.14	50.80 ± 5.30*	52.79 ± 5.52*	**< 0.001**
LVIDS (mm)	30.86 ± 2.76	35.43 ± 4.45*	37.15 ± 4.58*	**< 0.001**
IVSD (mm)	8.77 ± 1.42	9.06 ± 1.35	11.91 ± 1.33^*#^	**< 0.001**
LVPWD (mm)	8.94 ± 1.53	9.23 ± 1.59	11.76 ± 1.30^*#^	**< 0.001**
LAD (mm)	34.80 ± 2.74	40.69 ± 5.15*	42.76 ± 4.97*	**< 0.001**
LVEDV (ml)	69.26 ± 10.37	94.77 ± 32.49*	106.85 ± 30.89*	**< 0.001**
LVESV (ml)	28.89 ± 5.70	44.31 ± 19.40*	57.03 ± 23.24*	**< 0.001**
LVEF (%)	58.23 ± 3.37	55.63 ± 8.92	52.67 ± 8.19*	**< 0.001**
E/A	1.15 ± 0.19	0.99 ± 0.15*	0.62 ± 0.18^*#^	**< 0.001**
E/e’	7.08 ± 1.36	8.86 ± 1.36*	12.22 ± 3.60^*#^	**< 0.001**

Data are expressed as mean ± SD. Bold number: *P* < 0.05. *P < 0.05, vs normal. ^#^*P* < 0.05, vs NLVH. LVIDD left ventricular internal diameter at end-diastole; LVIDS left ventricular internal diameter at end-systole; IVSD interventricular septal diameter; LVPWD left ventricular posterior wall diameter; LAD left atrial diameter; LVEDV left ventricular end-diastolic volume; LVESV left ventricular end-systolic volume; LVEF left ventricular ejection fraction; E/A: peak early diastolic velocity of mitral orifice/peak late diastolic velocity of mitral orifice (by pulsed Doppler); E/e’: peak early diastolic velocity of mitral orifice (by pulsed Doppler)/peak velocity during early diastolic of mitral annulus (by pulsed-wave tissue Doppler)

### Comparison of MW and strain parameters of participants

The NLVH and LVH groups had lower GWE and GLS but higher GWW than the control group (P < 0.05). The LVH group had lower GWE and GLS but higher GWW and PSD than the NLVH group (P < 0.05). In addition, the LVH group had significantly lower GWI and GCW than both the control and NLVH groups (P < 0.01), and had higher PSD than the control group. However, the control and NLVH groups had comparable GWI, GCW, and PSD (P > 0.05) (Table 3).

**Table 3:** MW and strain parameters for participants

	Controls (n = 35)	NLVH (n = 35)	LVH (n = 33)	*P* value
GWE (%)	96.20 ± 1.43	90.66 ± 4.19*	86.42 ± 4.63^*#^	**< 0.001**
GWI (mmHg%)	2137.46 ± 220.80	2171.34 ± 321.37	1781.18 ± 282.39^*#^	**< 0.001**
GCW (mmHg%)	2236.34 ± 238.44	2308.14 ± 339.81	1954.79 ± 275.42^*#^	**< 0.001**
GWW (mmHg%)	76.69 ± 30.60	199.26 ± 94.31*	256.64 ± 92.72^*#^	**< 0.001**
GLS (%)	-20.66 ± 1.78	-16.87 ± 2.70*	-14.67 ± 2.94^*#^	**< 0.001**
PSD (ms)	42.06 ± 9.43	59.97 ± 21.39	79.88 ± 18.45^*#^	**< 0.001**

Data are expressed as mean ± SD. Bold number: *P* < 0.05. *P < 0.05, vs. Normal. ^#^*P* < 0.05, vs. NLVH. GWE: global work efficiency; GWI: global work index; GCW: global constructive work; GWW: global wasted work; GLS: global longitudinal strain; PSD: peak strain dispersion

**Fig. 1:**
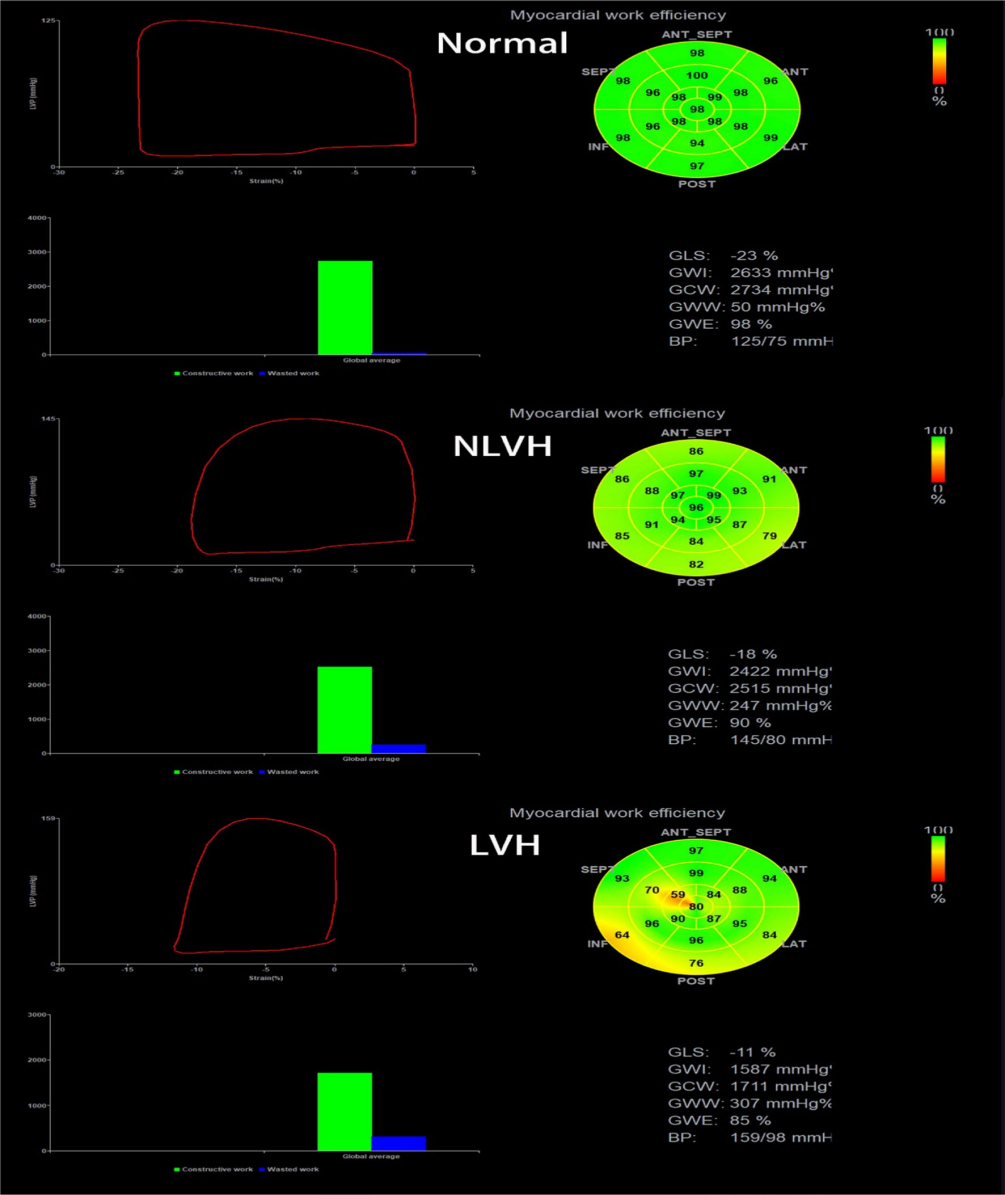
Comparison of left ventricular PSL and myocardial work parameters in control, NLVH, and LVH groups. The control group had the largest PSL area and good myocardial work parameters (GWE: 98%, GWW: 50 mmHg%, GWI: 2633 mmHg%, GCW: 2734 mmHg%). The NLVH group had a PSL area between those of the control and LVH groups, and some myocardial work parameters were poor (GWE: 90%, GWW: 247 mmHg%), while some were better (GWI: 2422 mmHg%, GCW: 2515 mmHg%). The LVH group had the smallest PSL area and worst myocardial work parameters (GWE: 85%, GWW: 307 mmHg %, GWI: 1587 mmHg%, GCW: 1711 mmHg%)

The control group had the largest PSL area and the smallest global average wasted work. The color of the bull’s eye in the control group was uniformly green, suggesting high work efficiency. The PSL area and global average wasted work for the NLVH group were between those for the control and LVH groups. The bull’s eye was unevenly light yellow, indicating a slightly lower work efficiency. The color of the bull’s eye in the LVH group was messy, with yellow and orange colors, indicating low work efficiency (Fig. 1).

### Definition of abnormal left ventricular function in LVH group

According to the diagnostic criteria for heart failure with preserved ejection fraction (HFpEF) proposed by the European Society of Cardiology in 2019 [14], the LVEF in the LVH group was normal or slightly reduced. However, these patients had cardiac function classes of II-IV according to the New York Heart Association (NYHA) cardiac function classification, and NT-proBNP > 125 pg/ml, GLS < 16%, LVH and/or left atrium enlargement, and diastolic dysfunction. Therefore, the patients in the LVH group had abnormal LV function.

### Correlation between MW parameters and LVEF in MHD with LVH patients

**Fig. 2:**
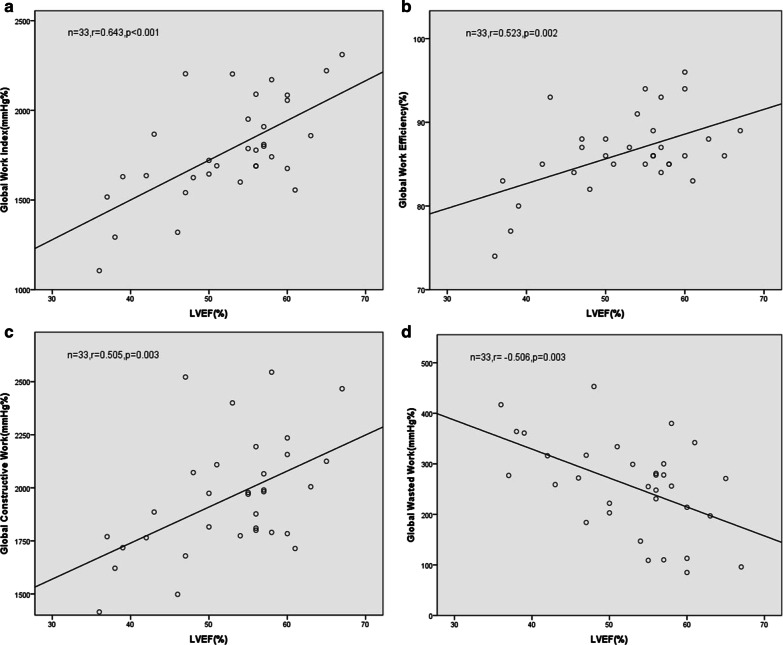
Analysis of correlation of the MW parameters GWI (**a**), GWE (**b**), GCW (**c**), and GWW (**d**) with LVEF in MHD with LVH patients. The correlation coefficients were 0.643, 0.523, 0.505, and − 0.506, respectively

We performed correlation analyses between the MW parameters and LVEF in the LVH group; the latter is a widely used indicator of cardiac function [15]. We found that LVEF was positively correlated with GWI, GWE, and GCW (r = 0.643, 0.523, 0.505; all P < 0.05), but negatively correlated with GWW (r =-0.506, P = 0.003) (Fig. 2).

### Determination of the cutoff values, sensitivity, and specificity of MW parameters and GLS for identifying LV function in MHD with LVH patients

**Fig. 3:**
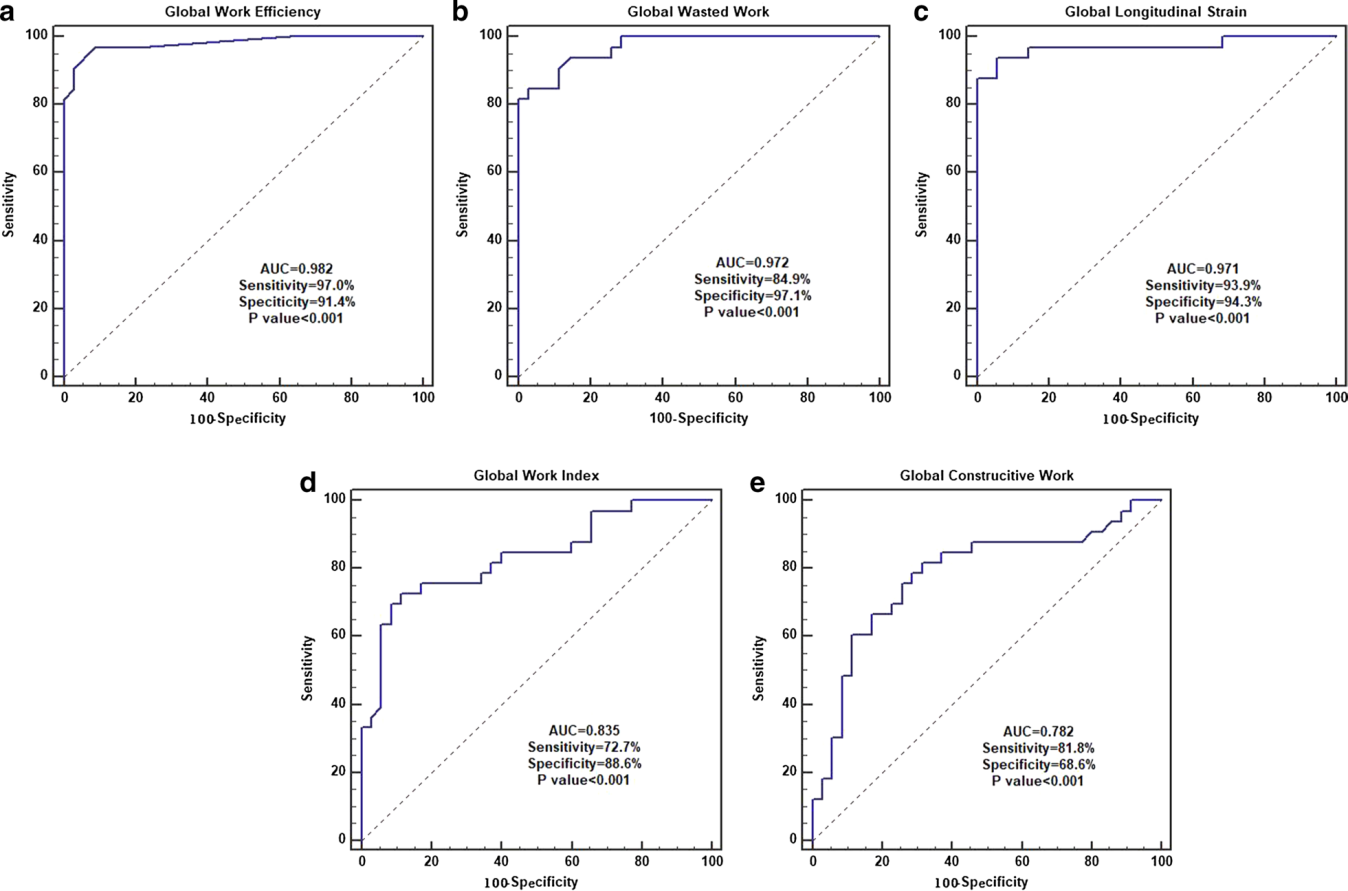
ROC curve analysis to evaluate the sensitivity and specificity of GWE (**a**), GWW (**b**), GLS (**c**), GWI (**d**) and GCW (**e**) for identifying LV dysfunction in MHD with LVH patients. The area under the ROC curve values for the GWE, GWW, GLS, GWI, and GCW were 0.982, 0.972, 0.971, 0.835, and 0.782, respectively. The sensitivity for the GWE, GWW, GLS, GWI, and GCW were 97.0%, 84.9%, 93.9%, 72.7%, and 81.8%, respectively. The specificity for the GWE, GWW, GLS, GWI, and GCW were 91.4%, 97.1%, 94.3%, 88.6%, and 68.6%, respectively. The cutoff values for the GWE, GWW, GLS, GWI, and GCW were 94%, 123 mmHg%, -18.44%, 1909 mmHg%, and 2157 mmHg%, respectively

We next used AUC curves to determine the sensitivity and specificity of MW parameters and GLS for identifying LV function in MHD with LVH patients. GWE, GWW, and GLS had AUCs of 0.982, 0.972, and 0.971, respectively (Fig. 3a, b, c), with respective cutoff values of 94%, 123 mmHg%, and − 18.44%. GWE had higher sensitivity than GLS and GWW (97.0% vs. 93.0% and 84.9%). In addition, GWW has higher specificity than GLS and GWE (97.1% vs. 94.3% and 91.4%). The AUCs for GWI (Fig. 3d) and GCW (Fig. 3e) were 0.835 and 0.782, respectively, both of which were lower than the AUCs for GWE, GWW, and GLS.

### Intra- and inter-observer variability

We also examined the intra- and inter-observer variability in this study. The intra-observer differences in the ICC values of GLS, PSD, LVEF, GWI, GCW, GWW, and GWE were 0.94, 0.93, 0.84, 0.86, 0.88, 0.96, and 0.88, respectively. The differences in the ICC values for these parameters between the observers were 0.93, 0.82, 0.79, 0.91, 0.89, 0.93, and 0.82, respectively. The ICC value and 95% confidence interval for each parameter are shown in Table 4. Our results suggested that our study generated reliable and consistent observations.

**Table 4:** Intra- and inter-observer variability

	Intra-observer		Inter-observer	
	ICC	95% CI	ICC	95% CI
GLS	0.94	0.89–0.97	0.93	0.85–0.96
PSD	0.93	0.86–0.97	0.82	0.66–0.91
LVEF	0.84	0.69–0.92	0.79	0.61–0.89
GWI	0.86	0.74–0.94	0.91	0.82–0.95
GCW	0.88	0.76–0.94	0.89	0.79–0.95
GWW	0.96	0.91–0.98	0.93	0.86–0.97
GWE	0.88	0.77–0.94	0.82	0.65–0.91

ICC, intra-class correlation coefficients; 95% CI, 95% confidence interval; GLS, global longitudinal strain; PSD, peak strain dispersion; LVEF, left ventricular ejection fraction; GWI, global work index; GCW, global constructive work; GWW, global wasted work; GWE, global work efficiency

## Discussion

In the present study, we examined the feasibility of MW parameters for evaluating the LV function in MHD patients with LVH and found that GWI, GCW, GWW, and GWE had appreciable AUC, specificity, and sensitivity in the LVH group. Therefore, we believe that these MW parameters may be reliably used to accurately evaluate the LV function in MHD patients, especially in those with LVH.

In this study, we first analyzed the conventional echocardiographic parameters for participants in the three groups and found that the MHD patients underwent LV remodeling, a structural basis for LV dysfunction [16, 17]. A previous study showed that there was no significant difference in LV diameter and wall thickness between non-hemodialysis and hemodialysis patients [18], indicating that hemodialysis does not significantly improve LV remodeling and cardiac contractile function in patients. On the contrary, with the prolongation of dialysis time and the aggravation of LV remodeling, the LV systolic function in MHD patients will be further impaired. The main feature of LV lesions in MHD patients is the progression of the compensatory phase of LVH to the decompensatory phase, because these patients have long-term pressure overload, including chronic hypertension, anemia, secondary hyperparathyroidism, and arteriovenous fistula [19, 20].

Our further analysis revealed that LVH and NLVH patients had significantly lower GLS than the controls. Moreover, with the increase in the LV thickness and prolongation of the dialysis time, the GLS decreased significantly, which was accompanied by a decrease in the LV systolic function, as evidenced by the lower GLS in MHD with LVH patients than in NLVH patients. These findings were in line with our previous report [21]. GLS is usually obtained from 2D-STI technology, which quantitatively measures the mechanical parameters for LV function [22, 23]. GLS is considered to be a better indicator than EF and is widely used in clinics to assess LV function [24]. However, some scholars recently suggested that strain measurements are susceptible to load, which in turn affects the accurate assessment of myocardial function [25]. Therefore, it is important to use effective screening methods to verify the GLS.

Recently, the MW parameters derived from PSL have been used to determine LV function and shown to have a good agreement between the noninvasively measured PSL and the invasively directly measured PSL [26]. In this study, we examined the possibility of using MW parameters to assess LV function in MHD patients. We found that the LVH group had significantly lower GLS, GWI, GCW, and GWE but higher GWW than the control and NLVH groups. LVH patients have pathological cardiac changes including disordered arrangement of myocardial fibers and increased myocardial interstitial fibrosis due to damage to myocardial cells [27]. In addition, LVH reduces the density of myocardial microcirculation and myocardial blood supply [28], resulting in decreased GWI and GCW. Early studies have shown that GWI has a strong correlation with myocardial glucose metabolism, basically reflecting the local myocardial oxygen consumption [29, 30]. Galli et al. found that patients with ischemic or dilated cardiomyopathy had increased GWI and GCW after cardiac resynchronization therapy [31, 32]. Thus, GWI and GCW can be used as indicators of myocardial survival. It is noteworthy that GCW is also the main predictor of LV fibrosis [33].

In MHD with LVH patients, the LV myocardium is significantly damaged by the accumulated metabolites and increased myocardial fibrosis, resulting in the impairment of LV systolic function. In the normal heart, all myocardial segments contract in a synchronized manner, but MHD with LVH patients and those with myocardial fibrosis have altered electrophysiological characteristics in the myocardium, which causes myocardial excitation-contraction uncoupling [34]. Thus, the LV contraction is not synchronized in MHD with LVH patients, as reflected by the increased PSD. Hence, some LV segments elongate during contraction, significantly wasting MW, which is reflected by increased GWW. The decrease in GWE probably derived from increased GWW and decreased GCW in MHD with LVH patients, indicating that LVH in MHD patients can cause a significant reduction in LV systolic function.

The NLVH group had comparable GWI and GCW to the control group, but higher GWI and GCW than the LVH group. Since the degree of LV myocardial impairment in the NLVH patients was smaller than that in the LVH patients, LV remodeling and fibrosis were not as severe as those in the LVH patients. Thus, it is understandable that NLVH patients had higher GWI and GCW than the LVH patients. MHD patients usually have poorly controlled hypertension [35]. In order to counteract the increased afterload during contraction [11], the heart pump function is compensated by shifting to a higher energy level [36], which is evidenced by higher GWI and GCW. This also suggests that the LV myocardium was not significantly impaired in NLVH patients and that LVEF was maintained at a normal level by enhanced myocardial contractility. However, if the high GWI persists and exceeds the compensatory capacity of the myocardium, LV remodeling and dysfunction will occur [37]. In addition, the NLVH group had higher GWW but lower GWE than the control group, with no significant difference in the LV PSD between the groups. This suggests that the GWW of the LV begins to increase while the LV thickness and synchronicity are still within the normal range in MHD patients. However, due to a slight increase in GCW, GWE decreased slightly. In other words, MW increased in the NLVH patients to maintain the contractile function at a normal level, but myocardial ineffective work also increased, which can potentially lead to impaired myocardial systolic function.

LVEF is the most commonly used evaluator for LV systolic function in the clinic [38]. In the present study, we found that LVEF was positively correlated with GWI, GCW, and GWE, and negatively correlated with GWW in the LVH group. LV segments of the normal heart contract synchronously, and the MW generated by the myocardium to overcome the afterload can be quantified using GWI and GCW, both of which have a positive effect on LV ejection [32]. However, when GWW is at a low level, GWE should be close to 100% [39]. As the thickness of the myocardium and the degree of myocardial injury increases in MHD patients, the decrease in LV systolic function is accompanied by an increase in GWW and a decrease in GWI, GCW, and GWE. MW can thus be used to quantitatively analyze the degree of LV injury in MHD with LVH patients, thus serving as a new sensitive indicator.

Our further analysis of the GLS and MW parameters for LVH patients revealed that the AUCs of GWE, GWW, and GLS were significantly higher than that of GWI and GCW. This observation was different from that in a previous study [40], in which the AUCs of GWI and GCW in CHD patients were higher than those of GLS, GWE, and GWW. This discrepancy may be explained by the different mechanisms underlying CHD and LVH in MHD patients. CHD is caused by the reduction or interruption of blood supply in the coronary arteries, leading to ischemia and necrosis of myocardial cells and early development of myocardial dysfunction [41]. This directly leads to a reduction in GWI and GCW. On the contrary, the damage from hemodialysis to the heart muscles is independent of the existence of coronary atherosclerosis [42]. Instead, the cardiac muscle damage in MHD patients is caused by the rapid changes in load [43], and the LV systolic function will only be significantly weakened during the decompensation period. Therefore, the test efficacy of GWI and GCW in MHD patients is relatively low. On the other hand, we found that the AUCs of GWE and GWW were higher than that of GLS. In addition, one of the main limitations of GLS is its load-dependence, and the increase in afterload can reduce the wall tension, leading to a misinterpretation of the true contractile function of the myocardium [40, 44]. However, for MW measurement, the non-invasive PSL integrates the afterload into the LV strain parameters, and thus MW is an optimization of the strain values [45]. Hence, the conclusions obtained from using non-invasive PSL are more comprehensive and objective.

## Limitations

The limitations of this study should be noted. First, this study only evaluated the global LV MW in MHD patients and the LV 17-segment MW parameters were not investigated. Second, MW technology mainly presents the effect of afterload on LV strain. The effect of pre-load on LV strain in MHD patients needs to be further investigated in the future. Third, this study was a single-center study with a small sample size. Thus, our conclusions should be corroborated in future studies performed in multiple centers with large cohorts.

## Conclusions

We report here that the MW technique is an optimized 2D-STI technique and that the MW parameters, GWI, GCW, GWW, and GWE, can be used to assess LV function in MHD patients, especially in those with LVH.

## Data Availability

The datasets used and/or analyzed during the current study are available from the corresponding author on reasonable request.

## Abbreviations

MW: Myocardial work
2D-STI: Two-dimensional speckle tracking imaging
MHD: Maintenance hemodialysis
LVH: Left ventricular hypertrophy
ESRD: End stage renal disease
NT-proBNP: N-terminal pro-brain natriuretic peptide
LVMI: Left ventricular mass index
GLS: Global longitudinal strain
PSD: Peak strain dispersion
PSL: Pressure-strain loops
GWI: Global work index
GCW: Global constructive work
GWW: Global wasted work
GWE: Global work efficiency
LVEF: Left ventricular ejection fraction
LVIDD: Left ventricular internal diameter at end-diastole
LVIDS: Left ventricular internal diameter at end-systole
IVSD: Interventricular septal diameter
LVPWD: Left ventricular posterior wall diameter
LAD: Left atrial diameter
LVEDV: Left ventricular end-diastolic volume
LVESV: Left ventricular end-systolic volume
